# Supplementary material for: Effect of Early Management on Pain and Depression in Patients with Pancreatobiliary Cancer: A Randomized Clinical Trial
Source: Cancers (Basel). 2019 Jan 11;11(1):79. doi: 10.3390/cancers11010079 (PMC6356375; doi:10.3390/cancers11010079)
Supplement: Supplementary file 1 [file cancers-11-00079-s001.pdf]

Supplementary Materials: Effect of Early Management on Pain and Depression in Patients with Pancreatobiliary Cancer: A Randomized Clinical Trial

Sang Myung Woo, Mi Kyung Song, Meeyoung Lee, Jungnam Joo, Dae Hyun Kim, Jong Heun Kim, Sung-Sik Han, Sang-Jae Park, Tae Hyun Kim and Woo Jin Lee

(A)

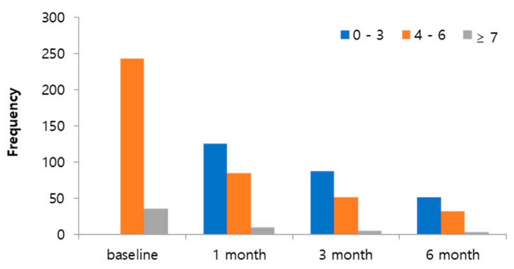

Time	Score		
	0 - 3	4 - 6	≥ 7
baseline	0	243	35
1 month	125	84	9
3 month	87	51	5
6 month	51	32	3

(B)

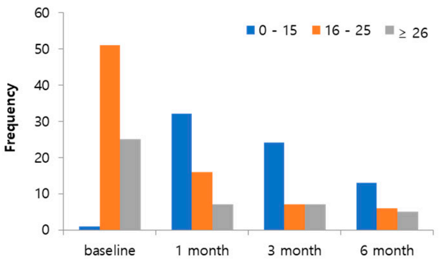

Time	Score		
	0 - 15	16 - 25	≥ 26
baseline	1	51	25
1 month	32	16	7
3 month	24	7	7
6 month	13	6	5

Figure S1. The distribution of BPI worst pain score (**A**) and CES-D (**B**).

Table S1. Baseline characteristics of patients who received chemotherapy.

Variables	Total Data Set (*n* = 201)		
	EPC	UOC	*p*-Value
**First-line chemotherapy**			0.7107
1 FOLFIRINOX: Fluorouracil + Irinotecan + Oxaliplatin	18 (18.56)	17 (16.35)	
2.Gemcitabine+Erotinib	32 (32.99)	33 (31.73)	
3.Gemcitabine+cisplatin	25 (25.77)	34 (32.69)	
4.Gemcitabine+abraxane	1 (1.03)	0 (0.00)	
5. Gemcitabine only	12 (12.37)	14 (13.46)	
6. other	9 (9.28)	6 (5.77)	
**Best Overall Response**			0.5973
Complete Response	0 (0.00)	0 (0.00)	
Partial Response	14 (14.43)	17 (16.35)	
Stable Disease	29 (29.90)	38 (36.54)	
Progressive Disease	25 (25.77)	26 (25.00)	
Not Evaluable	28 (28.87)	23 (22.12)	
Not Applicable	1 (1.03)	0 (0.00)	
**Nerve Block**			0.2428
No	81 (83.51)	80 (76.92)	
Yes	16 (16.49)	24 (23.08)	
**Operation**			0.2528
No	86 (88.66)	97 (93.27)	

Yes	11 (11.34)	7 (6.73)	
**Radiotherapy**			0.4367
No	76 (78.35)	86 (82.69)	
Yes	21 (21.65)	18 (17.31)	

Results reported as number (%).

Table S2. Quality of life for all patients 4 weeks after enrollment.

Group	Baseline	4 Weeks	*p*-Value †	Diff	*p*-Value ‡
Global health status					
EPC	50.00 (8.33–83.33)	50.00 (0.00–91.67)	0.0401	8.33 (−50.00–58.33)	0.0898
UOC	50.00 (0.00–100.00)	50.00 (16.67–100.00)	0.5893	0.00 (−66.67–83.33)	
Physical functioning					
EPC	73.33 (6.67–100.00)	73.33 (6.67–100.00)	0.1536	0.00 (−66.67–40.00)	0.0561
UOC	80.00 (0.00–100.00)	73.33 (0.00–100.00)	<0.0001	−6.67 (−60.00–86.67)	
Role functioning					
EPC	66.67 (0.00–100.00)	66.67 (0.00–100.00)	0.3933	0.00 (−83.33–66.67)	0.0140
UOC	66.67 (0.00–100.00)	50.00 (0.00–100.00)	0.0060	0.00 (−83.33–100.00)	
Emotional functioning					
EPC	79.17 (0.00–100.00)	83.33 (0.00–100.00)	0.1912	0.00 (−91.67–83.33)	0.3008
UOC	83.33 (8.33–100.00)	83.33 (0.00–100.00)	0.8178	0.00 (−66.67–58.33)	
Cognitive functioning					
EPC	83.33 (33.33–100.00)	83.33 (0.00–100.00)	0.2715	0.00 (−83.33–50.00)	0.3873
UOC	83.33 (0.00–100.00)	83.33 (16.67–100.00)	0.5731	0.00 (−50.00–83.33)	
Social functioning					
EPC	66.67 (0.00–100.00)	66.67 (0.00–100.00)	0.0625	0.00 (−100.00–66.67)	0.7298
UOC	66.67 (0.00–100.00)	66.67 (0.00–100.00)	0.0412	0.00 (−66.67–100.00)	
Fatigue					
EPC	33.33 (0.00–88.89)	33.33 (0.00–100.00)	0.9745	0.00 (−55.56–100.00)	0.0130
UOC	33.33 (0.00–100.00)	33.33 (0.00–100.00)	0.0005	11.11 (−100.00–88.89)	
Nausea and Vomiting					
EPC	0.00 (0.00–66.67)	0.00 (0.00–100.00)	0.0032	0.00 (−50.00–83.33)	0.1952
UOC	0.00 (0.00–100.00)	0.00 (0.00–100.00)	0.1043	0.00 (−100.00–100.00)	
Pain					

EPC	33.33 (0.00–100.00)	33.33 (0.00–100.00)	<0.0001	−16.67 (−83.33–50.00)	0.1773
UOC	33.33 (0.00–100.00)	33.33 (0.00–100.00)	<0.0001	−16.67 (−100.00–83.33)	
Dyspnea					
EPC	0.00 (0.00–100.00)	0.00 (0.00–100.00)	0.0606	0.00 (−100.00–100.00)	0.1825
UOC	0.00 (0.00–66.67)	0.00 (0.00–100.00)	0.0001	0.00 (−66.67–66.67)	
Insomnia					
EPC	33.33 (0.00–100.00)	33.33 (0.00–100.00)	0.0280	0.00 (−66.67–100.00)	0.4387
UOC	33.33 (0.00–100.00)	33.33 (0.00–100.00)	0.0760	0.00 (−100.00–100.00)	
Appetite loss					
EPC	50.00 (0.00–100.00)	33.33 (0.00–100.00)	0.3818	0.00 (−100.00–66.67)	0.3255
UOC	33.33 (0.00–100.00)	33.33 (0.00–100.00)	0.9562	0.00 (−100.00–100.00)	
Constipation					
EPC	33.33 (0.00–100.00)	33.33 (0.00–100.00)	0.9863	0.00 (−100.00–100.00)	0.5843
UOC	33.33 (0.00–100.00)	33.33 (0.00–100.00)	0.3393	0.00 (−100.00–100.00)	
Diarrhea					
EPC	0.00 (0.00–100.00)	0.00 (0.00–66.67)	0.8365	0.00 (−100.00–66.67)	0.5871
UOC	0.00 (0.00–100.00)	0.00 (0.00–66.67)	0.1318	0.00 (−100.00–66.67)	
Financial difficulties					
EPC	33.33 (0.00–100.00)	33.33 (0.00–100.00)	0.5741	0.00 (−66.67–66.67)	0.5167
UOC	33.33 (0.00–100.00)	33.33 (0.00–100.00)	0.1486	0.00 (−66.67–66.67)	

Results reported as median (range). †: Wilcoxon signed-rank test, ‡: Mann-Whitney U test.

Table S3. Mean (SD) of QLQ-C30 change scores in the three anchor-defined groups and the difference in mean change scores (95% CI) between adjacent categories. The number of patients in the anchor-defined groups varies by the scale and is therefore presented as a range of values for all the scales. Difference in mean change refers to the difference in mean of QLQ-C30 change scores between the "improvement" and "no change" (improvement) and between the "no change" and "deterioration" (deterioration).

QLQ-C30	Improvement	No change	Deteriorated	MID, 95% CI (95%)	
				Improvement	Deteriorated
Global health status	13.67 ± 18.14	5.30 ± 19.34	−4.84 ± 23.55	8.37 (4.84, 11.9) *	−10.14 (−15.11, −5.17) *
Physical functioning	5.87 ± 18.49	−3.88 ± 22.65	−13.76 ± 27.1	9.75 (5.85, 13.65) *	−9.88 (−15.63, −4.13) *
Role functioning	20.00 ± 31.55	−0.61 ± 29.39	−8.06 ± 33.57	20.61 (14.88, 26.34) *	−7.45 (−14.67, −0.23) *
Emotional functioning	17.67 ± 26.06	3.33 ± 24.35	−8.60 ± 30.69	14.34 (9.6, 19.08) *	−11.93 (−18.35, −5.51) *
Cognitive functioning	−4.00 ± 23.21	−2.73 ± 21.22	−4.84 ± 28.61	−1.27 (−5.45, 2.91)	−2.11 (−7.99, 3.77)
Social functioning	17.33 ± 21.77	−2.12 ± 21.29	−25.81 ± 26.82	19.45 (15.4, 23.5) *	−23.69 (−29.3, −18.08) *
Fatigue	−11.56 ± 23.67	4.24 ± 26.92	6.45 ± 35.81	−15.8 (−20.58, −11.02) *	2.21 (−5.18, 9.6)
Nausea and Vomiting	2.67 ± 22.40	12.12 ± 28.59	4.30 ± 23.16	−9.45 (−14.3, −4.6) *	−7.82 (−13.49, −2.15) *

Pain	−20.00 ± 27.22	−14.55 ± 28.34	−13.98 ± 29.22	−5.45 (−10.68, −0.22) *	0.57 (−5.92, 7.06)
Dyspnea	5.33 ± 28.35	4.85 ± 24.36	4.30 ± 35.22	0.48 (−4.48, 5.44)	−0.55 (−7.68, 6.58)
Insomnia	−20.00 ± 31.91	−4.85 ± 36.53	−7.53 ± 41.01	−15.15 (−21.62, −8.68) *	−2.68 (−11.54, 6.18)
Appetite loss	−16.00 ± 43.16	−5.45 ± 47.04	2.15 ± 42.11	−10.55 (−19.06, −2.04) *	7.6 (−2.27, 17.47)
Constipation	2.67 ± 40.73	0.00 ± 35.14	−2.15 ± 38.43	2.67 (−4.48, 9.82)	−2.15 (−10.52, 6.22)
Diarrhea	−1.33 ± 17.95	2.42 ± 26.34	−1.08 ± 31.6	−3.75 (−8.01, 0.51)	−3.5 (−10.2, 3.2) *
Financial difficulties	−37.33 ± 11.06	0.00 ± 0.00	35.48 ± 8.32	−37.33 (−38.78, −35.88) *	35.48 (33.99, 36.97) *

* Differences that are statistically significant.

© 2019 by the authors. Submitted for possible open access publication under the terms and conditions of the Creative Commons Attribution (CC BY) license (http://creativecommons.org/licenses/by/4.0/).
